# The search for rigid, tough polyesters with high *T*_g_ – renewable aromatic polyesters with high isosorbide content†

**DOI:** 10.1039/d4su00294f

**Published:** 2024-08-01

**Authors:** Bruno Bottega Pergher, Daniel H. Weinland, Robert-Jan van Putten, Gert-Jan M. Gruter

**Affiliations:** a Industrial Sustainable Chemistry, van't Hoff Institute of Molecular Sciences, University of Amsterdam Science Park 904 1098 XH Amsterdam The Netherlands G.J.M.Gruter@uva.nl; b Avantium Support B.V. Zekeringstraat 29 1014 BV Amsterdam The Netherlands

## Abstract

Renewable polyesters with a good balance between impact strength and elastic modulus (stiffness) are not very common, especially when combined with high glass transition temperature (*T*_g_). Achieving such high performance properties would enable the substitution of high performance polymers like ABS and polycarbonate with chemically recyclable polyesters from bio-based or recycled sources. One of the challenges in developing these materials is to select the right composition of the right monomers/comonomer ratios and making these materials with high molecular weight, which can be challenging since some of the most promising rigid diols, such as isosorbide, are unreactive. This study comprises aromatic polyesters from (potentially) renewable monomers, using bio-based isosorbide as a means to increase their *T*_g_ and to inhibit their crystallization, while using flexible co-diols to improve impact strength. To incorporate a high amount of isosorbide into the targeted polyesters, we used the synthesis method with reactive phenolic solvents previously developed in our group. The selected compositions display high *T*_g_'s (>90 °C) and high tensile modulus (>1850 MPa). We show that more polar monomers such as the stiffer 2,5-furandicarboxylic acid (FDCA) and diethylene glycol cause high stiffness but decreased impact strength (<5 kJ m^−2^). Combining terephthalic acid and isosorbide with more flexible diols like 1,4-butanediol, 1,4-cyclohexanedimethanol (CHDM) and 1,3-propanediol provides a better balance, including the combination of high tensile modulus (>1850 MPa) and high impact strength (>10 kJ m^−2^).

## Abstract 2

Renewable polyesters with a good balance between impact strength and elastic modulus (stiffness) are not very common, especially when combined with high glass transition temperature (*T*_g_).

## Graphical abstract

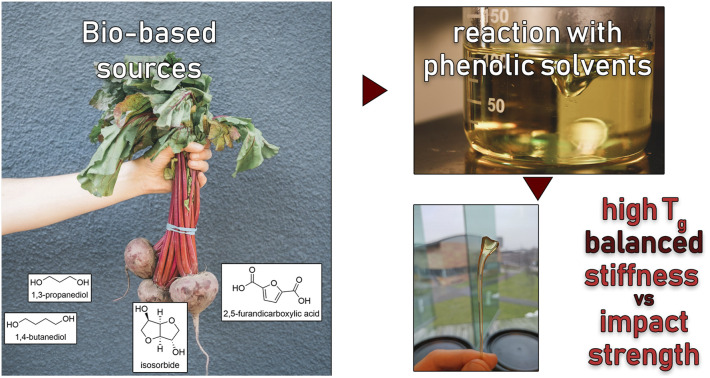

**Sustainability spotlight**

To realize the goal of a net-zero world, sustainable and circular (closed-loop recyclable) plastics are very important. Polyolefins such as polyethylene and polypropylene and fossil copolymers such as ABS (acrylonitrile-butadiene-styrene) have poor carbon footprints and are not or at best poorly recyclable. Chemo-catalytic recycling to transform waste plastics *via* pyrolysis and hydrotreating are high cost, energy intensive and require high operating temperatures and have low atom efficiency. We are developing (novel) polyesters from renewable resources (bio-mass, CO_2_ or waste plastics) that can compete with fossil analogs on performance and (at scale) on production cost. Renewable polyesters with a good balance between impact strength and elastic modulus (stiffness) are not very common, especially when combined with high glass transition temperature (*T*_g_) for enhanced ageing properties. Achieving such high performance properties enables the substitution of high performance polymers like ABS and polycarbonate with chemically recyclable polyesters from bio-based or recycled sources. Such copolyesters are not easy to produce. To incorporate a high amount of rigid diol into the targeted polyesters, we used a novel synthesis method with reactive solvents previously developed in our group. Our work emphasizes the importance of the following UN sustainable development goals: industry, innovation, and infrastructure (SDG 9), responsible consumption and production (SDG 12) and climate action (SDG 13).

## Introduction

Polymers with a good balance between impact strength, stiffness and glass transition temperature are required for applications such as protective screens, LEGO bricks, car parts, musical instruments *etc.* Those applications require polymers such as acrylonitrile butadiene styrene (ABS) and bisphenol A polycarbonate (PC), both fossil based, which display high *T*_g_, stiffness and toughness. Given the large market size of such polymers, the development of alternatives which are more easily (chemically) recyclable *via* hydrolysis or alcoholysis and sourced from renewable feedstock could have a significant impact. In terms of material usage and sourcing, polyesters present two main advantages. Firstly their monomers can potentially be produced from renewable sources, such as biomass, CO_2_, and from chemically recycled polyesters such as terephthalic acid and ethylene glycol from PET. Biomass and CO_2_ are good feedstock candidates for polyester monomer synthesis because their inherent high oxygen content matches the composition needs of polyesters. Using these carbohydrate resources to produce polymers that are poor in oxygen (such as bio-polyethylene and bio-polypropylene) would imply a considerable loss of mass (at least 3.2 tons of glucose per ton ethylene or propylene) resulting in a considerably higher feedstock cost.^1^ The second advantage is that, after the polyesters are produced and used, their ester bonds can be hydrolysed and the polyesters can be chemically recycled into their monomers and ester derivatives, which can be purified, allowing for the production of closed-loop recycled materials of virgin quality. Therefore, the development of new polyesters with high rigidity (modulus, *E*), high impact strength (*σ*) and high *T*_g_ could play an important part in the use of (potentially) renewable materials in high performance applications and provide alternatives to polymers like ABS.

Achieving high stiffness and high impact strength in a single polyester is a challenge, especially when combined with high glass transition temperature (*T*_g_). Many of the commercial polyesters possess either high stiffness, like poly(ethylene terephthalate) (PET) and poly(lactic acid) (PLA), or else high impact strength, like poly(1,4-cyclohexanedimethanol terephthalate) (PCT), PCTG (PCT modified with ethylene glycol)^2,3^ and poly(1,4-cyclohexanedimethanol-*co*-2,2,4,4-tetramethyl-1,3-cyclobutanediol terephthalate) (PCTT, commercially known as *Tritan*). Out of all of these materials, only *Tritan* has a *T*_g_ above 100 °C.

A significant number of monomers are already commercially produced from sugars, which facilitates the search for alternatives to fossil-based materials. Some of these monomers are studied in this paper: 1,4-butanediol,^4^ 1,3-propanediol,^5^ isosorbide,^6^ succinic acid,^7^ and 2,5-furandicarboxylic acid (FDCA),8^8^ are all obtainable *via* glucose (either directly or *via* fructose). Glucose as a feedstock for materials should, ideally, come from lignocellulosic sources, like agricultural residues, as to not compete with food sources or land use, though the production of lignocellulosic glucose is not yet implemented at industrial scale. Terephthalic acid (or derivatives like dimethyl terephthalate, DMT or bis(2-hydroxymethyl) terephthalate, BHET) and 1,4-cyclohexanedimethanol (CHDM) can more feasibly be produced *via* the chemical recycling of PET,^9,10^ but likewise this pathway is not yet used at very large scale.

Isosorbide is an important component for renewable high performance polyesters, given that it can be used for improving *T*_g_ and stiffness in polymers. Furthermore, it can serve to disrupt the material's crystallinity, typically when it makes up more than 30% of the diol content. The reactivity of isosorbide is limited, however, because it has two secondary hydroxyl groups. To overcome this issue, our group developed a strategy^11,12^ to synthesize high molecular weight polyesters with isosorbide in high content, which enables the production of renewably sourced polyesters like poly(isosorbide succinate), PIsSu. This method makes use of reactive solvents like *p*-cresol, which results in the production of unprecedentedly high molecular weights for some polyesters. This method opened up the possibility of producing high molecular weight polyesters with *T*_g_'s significantly over 100 °C. When isosorbide is used as the only diol, however, in combination with rigid diacids, the resulting polymers will be typically very stiff. This which can be countered by partially replacing isosorbide by other diol co-monomers.

Combining “stiff” and “flexible” co-monomers is a strategy to balance the properties of a material. Such is the case for improving the impact properties of furanoate polyesters, which are usually very stiff due to the structure of 2,5-furandicarboxylic acid (FDCA).^13^ Other methods include blending of polymers like PET with (impact) modifiers such as elastomers and/or glass fibre, which can drastically improve PET's impact properties but may reduce stiffness in the case of added elastomers and will add more components, potentially fossil-based, which makes (chemical) recycling more challenging.^14,15^ This research focuses on new materials that may possess a good balance between stiffness, impact strength and *T*_g_. In particular we explore the use of isosorbide as a stiff, biobased monomer in balance with flexible diol co-monomers, producing high molecular weight polyesters with high *T*_g_, relatively high tensile modulus and high impact strength.

## Experimental

### Materials

Terephthalic acid (≥99%), 1,4-butanediol (>99%), 4-methyl phenol (>99%), 4-ethyl phenol (98%), 4-methoxyphenol (99%) were purchased from Acros Organics. Diethylene glycol (99%), dimethyl terephthalate (≥99%), *p*-methoxyphenol (99%) and butyltin hydroxide oxide hydrate (97%) were purchased from Sigma-Aldrich. 1,4-Cyclohexanedimethanol (>99%) was purchased from TCI, with 70% of the trans isomer. Isosorbide was acquired from Roquette Frères (≥99.9%). 1,3-Propanediol (99%) is from Alfa Aesar. The “PET” studied was *RamaPET N180* from Indorama Ventures. “Tritan” (TX1001) was obtained from Eastman, and “ABS” (Terluran GP-35) from INEOS Styrolution.

2,5-Furandicarboxylic acid (>99.5%) was provided by Avantium.

Bisphenol A “Polycarbonate” was Lexan 144R from Sabic.

Some of the values reported in the tables were obtained from the literature, therefore not from using the aforementioned materials. In those cases, the original literature reference is provided.

### Synthesis method

All polyesters were synthesized in a two-step method with phenolic reactive solvents present, according to the synthesis strategy described previously by our group,^11,12^ using a three-necked round-bottom flask of 100 mL. Typical experiments were performed with 10–25 g of diacid, and diols were added such that the molar ratio between total diols and diacids was between 1.0 and 1.1. The diacid, diols, reactive solvent and catalyst (butyltin hydroxide oxide hydrate, 0.14 mol% relative to the diacid) were all added to the reaction flask from the start, in the amounts shown in Table 1. The reaction vessel was then heated in an oil bath and kept under nitrogen flow at 40 mL min^−1^, and stirred at 100 rpm. The heating temperature was kept high enough to keep the solvent boiling without distilling out. The reaction progression was followed by taking samples which were analysed with ^1^H-NMR.

**Table 1:** Synthesis parameters and characterization of the polyesters produced in this study *via* reactive solvent synthesis

Material^a^	Polymer composition IS/co-diol [feed]^b^ (mol%)	*t*_PC_ (h)	Solvent^c^ {equiv.}	*T*_est._ (solv. bp)^d^/*T*_PC_ (°C)	*M*_*n*_ (kg mol^−1^) {PDI}^e^	Sample type
PIT	97.8/0.0 [100/0]	2.0	MP {1.2}	260 (243)/260–310	12.3 {2.6}	Tensile, *T*_g_
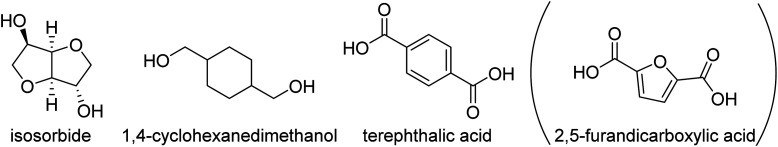						
PI_40_CT	39.9/60.7 [43/60]	0.5	MP {1.2}	264 (243)/299	16.5 {2.3}	Tensile
	41.0/58.8 [43/60]	2.5	Cre {1.2}	240 (202)/275	24.8 {1.9}	Impact, *T*_g_
PI_50_CT (“PICT”)	51.1/48.9 [53/50]	1.0	EP {1.2}	260 (218)/282	19.2 {2.2}	Tensile, *T*_g_
	49.8/49.6 [53/50]	1.2	EP {1.0}	250 (218)/280	20.0 {2.1}^i^	Impact
PI_60_CT	60.9/41.2 [63/40]	0.6	MP {0.9}	260 (243)/295	18.7 {2.2}	Tensile
	59.7/38.9 [60/40]	3.0	Cre {1.2}	260 (202)/285	23.8 {2.4}	Impact, *T*_g_
PI_40_CTF_10_^h^	41.1/60.0 [43/60]	2.0	Cre {1.2}	240 (202)/280	28.8 {1.9}	Tensile
	39.6/59.9 [40/60]	2.0	Cre {1.2}	240 (202)/270	42.0 {2.1}	Impact, *T*_g_
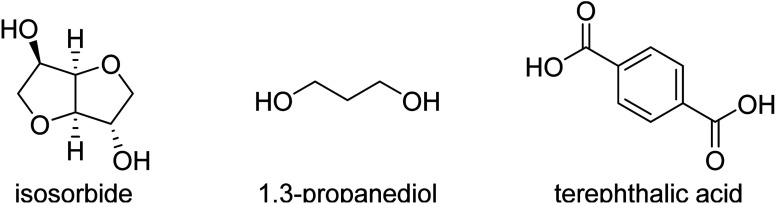						
PI_40_PT	39.6/60.1 [40/62]	1.0	EP/MP^f^ {1.2}	240 (233)/280	21.7 {2.2}	Tensile
	40.0/60.2 [40/60]	2.0	Cre {1.2}	235 (202)/270	21.1 {2.0}	Impact, *T*_g_
PI_50_PT (“PIPT”)	50.0/50.0 [50/50]	2.5	Cre {1.2}	230 (202)/270	25.4 {2.0}	Tensile, *T*_g_
	50.1/49.8 [50/50]	1.5	EP {1.2}	250 (202)/275	23.3 {2.1}	Impact
PI_60_PT	60.4/39.8 [60/40]	1.5	EP {1.2}	245 (218)/270	19.2 {2.1}	Tensile, impact, *T*_g_
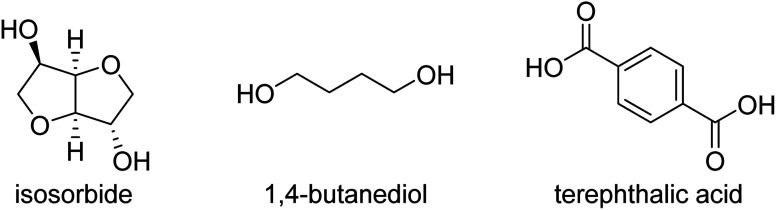						
PI_50_BT (“PIBT”)	52.7/48.2 [55/55]	3.0	Cre {1.0}	230 (202)/280	17.8 {2.1}	Tensile, *T*_g_
	53.6/45.8 [55/55]^g^	3.0	Cre {1.0}	230 (202)/280	17.9 {2.1}	Impact, *T*_g_
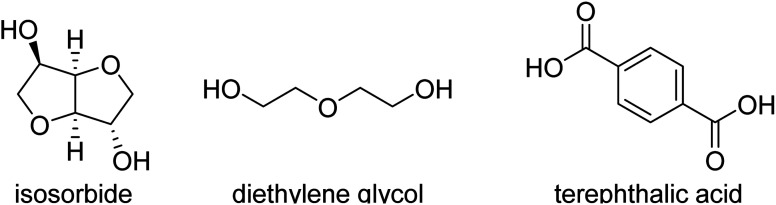						
PI_50_DT (“PIDT”)	50.0/50.0 [53/50]	4.0	Cre {1.2}	230 (202)/280	25.9 {2.0}	Tensile, *T*_g_
	50.0/50.0 [53/50]	4.0	Cre {1.2}	230 (202)/280	20.1 {2.3}	Impact
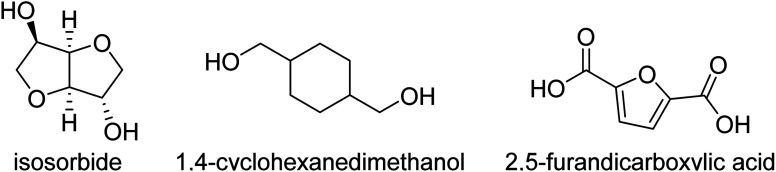						
PI_50_CF (“PICF”)	50.0/50.0 [50/50]	0.6	MP {1.5}	230 (243)/265	23.5 {2.1}	Tensile
	50.0/50.0 [50/50]	1.5	Cre {1.2}	230 (202)/260	29.9–37.0 {1.8}	Impact, *T*_g_
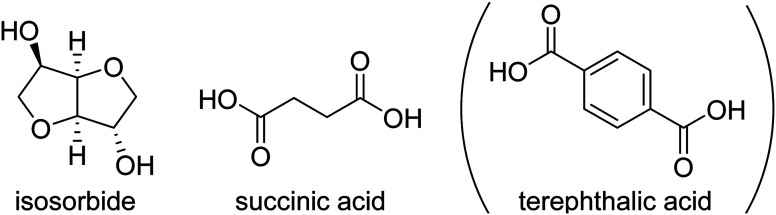						
*PIsSu* j	*100.0/0.0 [100/0]*	*1.0*	*Cre {1.5}*	*240 (202)/220*	*35.3 {2.1}*	*Tensile, T*_*g*_
	*100.0/0.0 [100/0]*	*1.8*	*Cre {1.5}*	*240 (202)/220*	*43.9 {2.7}*	*Impact*
PIsSuT_25_^h^	100.0/0.0 [100/0]	2.0	Cre {1.5}	250 (202)/225	20.2 {2.5}	Tensile, *T*_g_
	100.0/0.0 [100/0]	4.0	Cre {1.5}	240 (202)/220	25.1 {2.5}	Impact
PIsSuT_33_^h^	100.0/0.0 [100/0]	2.3	Cre {1.5}	250 (202)/230–250	17.2 {2.4}	*T*_g_

a Subscript after “I” indicates mol% of isosorbide (*vs.* total diol) present in polymer as quantified by NMR. *PIsSu*, poly(isosorbide succinate) was produced previously in our group.^11^ The letters represent the following: “I” or “Is” for isosorbide, “C” for CHDM, “T” for terephthalate, “P” for 1,3-propanediol, “B” for 1,4-butanediol, “D” for diethylene glycol, “F” for 2,5-furandicarboxylate and “Su” for succinic acid. All materials without explicitly indicated isosorbide content have ∼50 mol% isosorbide (relative to total diol) – PICT, PIBT, PIPT, PIDT, PICF.

b Composition of co-monomers, in mol%, relative to the diacid peak. The first values are quantified from the polymer *via*^1^H-NMR, while the values in square bracket are the values used in the feed. Total diol content from NMR in some cases shows deviation from the expected 100% – this is due to the inaccuracy of NMR calculations due to for example weak end group signals and potential signal overlap.

c Reactive solvents used for synthesis: 4-ethyl phenol (EP), 4-methoxy phenol (MP), 4-methyl phenol *i.e.* cresol (Cre).

d *T*_est_ is the temperature of the oil bath used during esterification, while the boiling point of the solvent is displayed between brackets. *T*_PC_ is the oil bath temperature during polycondensation.

e Number-average molecular weights from batch(es) used for tensile test and batches for impact test (in square brackets).

f Mixture of EP and MP, 1 : 1 in volume.

g Batch for impact synthesized using Ti(OBu)_4_ as catalyst, at 0.05 mol% (*vs.* diacid).

h PI_40_CTF_10_ contains, in diacid content, 90 mol% terephthalic acid and 10 mol% FDCA. Similarly, PIsSuT_25_ contains 75 mol% succinic acid and 25 mol% terephthalic acid. *T*_g_ of PIsSuT_33_ is 109 °C – not shown in Table 2 because its mechanical properties have not been evaluated.

i Batch produced in our 2 liter autoclave.

j Batch produced by Weinland.^11^

After the first step (esterification) was deemed finished (6–14 h), gradual vacuum was applied to the system (down to ∼0.5 mbar, typically within 1.5 h), and the temperature of the oil bath was typically increased to at least 260 °C (up to 310 °C; see Table 1). Once a pressure of ∼0.5 mbar was reached, polycondensation (PC) was considered to have started, at which point very little solvent (unreacted or as end-groups) remained in the system. The polycondensation was performed either for a fixed amount of time (*t*_pc_) or the step was finalized based on torque stagnation and distillation temperature stabilization.

One batch of polyester was produced in a 2 liter autoclave (Büchi AG, type 3), for which the reaction parameters are also provided in Table 1.

### Characterization

GPC was used for obtaining molecular weight data. A LC column from Agilent (Poroshell 120 EC-C18, 4.6 × 100 mm, 2.7 μm) was used with dichloromethane (DCM, at 35 °C) as the eluent, with polystyrene standards. We used the data from a refractive index (RI) detector (1260 Infinity II).

^1^H-NMR was performed on a Bruker AMX 400 (400 MHz) using as solvent deuterated chloroform (CDCl_3_). The molar quantities (mol%) shown in Table 1 are calculated in relation to the terephthalate or furanoate peak.

The glass transition temperatures were recorded from the second heating cycle on a differential scanning calorimeter (DSC) from Mettler Toledo (DSC 3+ STARe). The program applied cycles at a rate of 10 °C min^−1^ (heating) and −10 °C min^−1^ (cooling). They were performed from 25 °C to 260 °C or 300 °C, depending on the *T*_g_ (all polyesters reported were amorphous), with nitrogen gas flow (50 mL min^−1^).

Thermogravimetric analysis (TGA/DSC 3+ STARe, from Mettler Toledo) was conducted to analyse the thermal stability of the polyesters. Different programs were used: heating ramps at 10 °C min^−1^, from 25 °C to 600 °C, under nitrogen gas flow at 50 mL min^−1^, and isotherms at *T*_injection_, *i.e.* the temperature used to melt that material for injection moulding, under N_2_ gas flow or air flow (50 mL min^−1^), and isotherms at 280 °C under nitrogen flow (50 mL min^−1^). The isotherms were heated at 30 °C min^−1^ to their fixed final temperature, and kept at that temperature for 120 minutes.

Samples for mechanical testing – impact and tensile – were produced *via* injection moulding, using a HAAKE MiniJet II, from Thermo Fischer Scientific. The tensile samples were produced according to ISO 527-2, sample type 5A, and the impact bars according to ISO 179, with v-notched samples of 80 × 10 × 4 mm^3^.

The tensile properties were assessed with an INSTRON Universal Testing System, measured at 5 mm min^−1^ (crosshead speed). The tensile modulus was measured within the strain range of 0.05% to 0.25%. The (offset) yield strength was measured using an offset of 0.2%, according to ASTM D638-03.

The impact strength was measured using a Zwick Pendulum Impact Tester using a Charpy hammer, with v-notched samples and according to ISO 179. Each sample set had at least 3 specimens, for both tensile and impact tests.

## Results and discussion

### Synthesis of polyesters

The materials synthesized in this study, together with their synthesis parameters, are shown on Table 1. These polyesters were produced at molecular weight ranges (*M*_*n*_ 18–42 kg mol^−1^) that are usually sufficient for various commercial applications. This is in line with previous studies from our group that showed the effectiveness of reactive phenolic solvents in the synthesis of high isosorbide content (IS%) polyesters.^11,12^ This work adds a new selection of important materials to the list, focusing on aromatic copolyester candidates for high impact, high rigidity applications. The literature presents polyesters equivalent or similar to some of the ones in this work, like PICT,^16–24^ PICF^25–27^ and PIBT,^28–30^ however, all with lower molecular weights and often lower isosorbide content. Apart from the advantage of higher molecular weights, our synthesis method typically requires little or no diol excess in the feed, which makes it easier to reach the target composition. The polyester with butanediol (PIBT) did, however, require excess diol due to side-reactions taking place (THF formation).

High isosorbide content (≥40%) was chosen for different reasons: above 40 mol% (*vs.* total diol), isosorbide likely hinders crystallization (ESI†), which is desired for processability, and because the chosen polyesters are likely to have high *T*_g_ (>100 °C) with high IS content. 50% isosorbide content was selected as a reference composition due to experimental results obtained during this study, which showed a good balance of properties for some of the polyesters, especially PICT. Higher (>60%) amounts of isosorbide, however, create difficulties in synthesis and processing due to increasingly high viscosities, besides possibly adding too much stiffness at the cost of impact strength.

### The delicate balance: elastic modulus (*E*), impact strength (*σ*) and *T*_g_

The mechanical properties of the synthesized materials, obtained from tensile and impact testing, together with their *T*_g_, are shown in Table 1. PICT and PIPT were studied in different compositions to evaluate how shifts in the amount of isosorbide and co-diol cause changes in polymer properties. An important goal of our studies is to find candidates that could potentially replace ABS, or even polycarbonate. For that purpose, materials were targeted with *T*_g_ > 100 °C, impact strength *σ* > ∼20 kJ m^−2^ and elastic modulus *E* > ∼2000 MPa. The studied materials exhibited significant differences depending on their isosorbide content and on the type of co-diol and diacid.

**Fig. 1:**
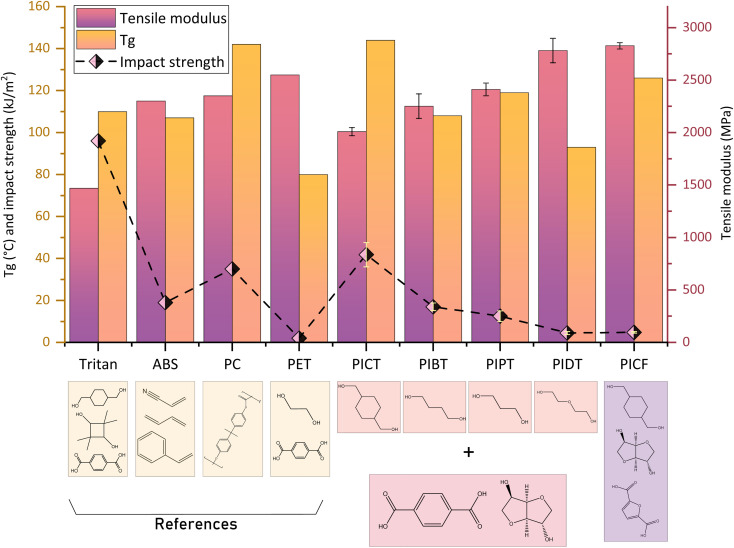
*T*_g_, impact strength and tensile modulus of different materials with ∼50% isosorbide content. The co-diols of isosorbide are: CHDM (C), 1,4-butanediol (B), 1,3-propanediol (P) or diethylene glycol (D).

Fig. 1 shows *T*_g_, impact strength and tensile modulus for the studied polyesters – in which 50 mol% of the diols is isosorbide and 50 mol% is a different co-diol for each case – alongside four references: ABS, BPA-based polycarbonate, PET and PCTT (commercially known as *Tritan*). The main reference for us, ABS, shows that balancing rigid components (acrylonitrile and styrene) with a rubbery phase (butadiene) allows for high *T*_g_ and relatively high impact strength and modulus. A similar balance is kept with polycarbonate, which has even higher *T*_g_ and impact strength. This balance is not kept with PET, for which the π–π stacking and the homogeneity of TPA and ethylene glycol promotes crystallinity and high cohesion between chains, resulting in high modulus but poor impact strength. PCTT benefits greatly from the presence of CHDM for its impact performance, though the two bulky diols make interchain interaction limited, resulting in a low value for tensile modulus. By synthesizing polymers with different diols, however, the mentioned properties can be tuned.

**Fig. 2:**
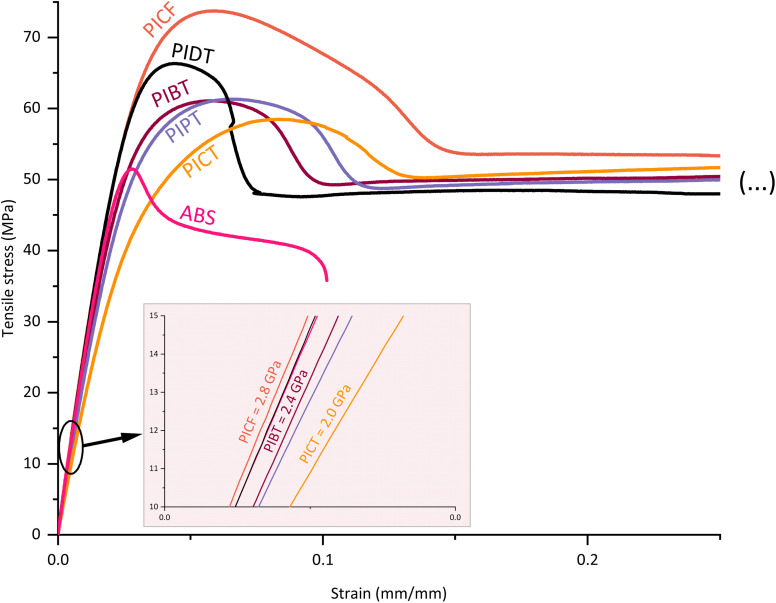
Tensile curves of the main synthesized co-polyesters with isosorbide.

The tensile test results indicate an increase in stiffness when using co-diols with low flexibility and mobility. Such is the case when comparing the more flexible butanediol polyester PIBT with the more rigid propanediol polyester PIPT (curves in Fig. 2). The flexibility and mobility of the co-monomers affect the mechanical properties of the polymer by influencing the capacity of the chains to absorb impact energy as molecular motion or as configuration shift, and by affecting the cohesion between chains and the (thermal) energy required for these movements. In general, shifting the composition of a polyester towards more rigid co-monomers will increase the tensile modulus at the expense of impact strength.^22,42^ It is necessary, therefore, to tune the compositions carefully according to the desired applications, finding a balance in performance parameters. The other tensile properties indicated in Table 2 show that the synthesized polyesters are ductile at the presented molecular weights – ductile behaviour for PICT for *M*_*n*_ > ∼13 kg mol^−1^ and > ∼20.0 kg mol^−1^ for PICF, for instance. The materials in this study elongate considerably before rupture (∼17–142%), display good yield strength (∼33–55 MPa), which allows the material to withstand load without permanent deformation, and have considerably high stiffness (tensile modulus ∼1850–2900 MPa). The differential amongst them is the impact strength.

**Table 2:** Mechanical properties and *T*_g_ of the synthesized polyesters. For the polymer abbreviations used please see legend of Table 1

Material	Tensile strength (MPa)	Yield strength (MPa)	Elongation at break (%)	Tensile modulus, “*E*” (MPa)	Impact strength, “*σ*” (kJ m^−2^)	*T*_g_ (°C)	Injection temperature (°C)
ABS	51	44	12	2300	19	107	—
Polycarbonate^31^ (PC)	∼63	∼57	110	2350	35	142	—
PET^32^	66	61	131	2550	∼2–3	∼76–80	—
PCTT (Tritan)	46	43	210	1550	93	110	—
PIT	61 [4.2]	35 [4.2]	9 [1.7]	2590 [55.7]^b^	—	188	280
PCT^33,34^	50–60	47	320	1400–1600	∼10	∼90	—
PI_40_CT	53 [1.5]	33 [1.0]	57 [20.7]	1850 [37.1]	56 [0.9]	133	260
PI_50_CT (PICT)	57 [1.0]	34 [0.9]	50 [11.8]	2010 [39.0]	42 [5.8]	144	255–265
PI_60_CT	60 [1.1]	35 [0.6]	17 [8.8]	2120 [18.1]	53^a^ [4.1]	153	270–275
PI_40_CTF_10_	57 [1.9]	35 [1.7]	73 [35.9]	2000 [120.1]	88 [3.7]	133	260–265
PPT^35–37^	50–60	60	15^d^	2000–2600	3–5	53	—
PI_40_PT	56 [1.1]	40 [1.7]	138 [20.6]	2890 [148.3]	10 [1.2]	103	220
PI_50_PT (PIPT)	60 [1.9]	41 [0.8]	121 [29.6]	2410 [61.2]	13 [2.5]	119	215–235
PI_60_PT	61 [1.0]	36 [3.3]	63 [40.5]	2620 [100.9]	10 [4.0]	130	240
PBT^33,38^	62–65	—	—	1400–1600	8.7	∼27	—
PI_50_BT (PIBT)	60 [1.0]	42 [0.9]	115 [35.7]	2250 [117.2]	17 [1.8]	108	220
PDT^39^	—	—	—	—	—	33	—
PI_50_DT (PIDT)	66 [0.9]	47 [3.1]	187 [2.4]	2780 [116.5]	5 [0.7]	94	215
PCF^40,41^^e^	56–63 [4]	—	19 [4] to ∼180	1350 [180], ∼2200	∼4.6 (Izod)	83	250–270
PI_50_CF (PICF)	74^a^ [0.8]	45^a^ [5.0]	85^a^^,^^b^^,^^c^ [71.6]	2830^c^ [31.1]	5 [0.4]	128	240–245
PIsSu^11^^f^	85 [2.0]	55 [1.4]	139 [82.0]	3720 [100.0]	3 [0.1]	80	180
PIsSuT_25_	71 [1.8]	53 [1.5]	55 [74.6]	3370 [42.8]	4 [0.4]	102	185

a Standard deviation of samples is shown in square brackets. Materials in italic were not synthesized, but their values were obtained from commercial documents and/or literature studies.

b Relatively brittle samples due to low MW.

c Only 2 samples used.

d Tensile test run at 50 mm min^−1^ (cross-head speed).

e PCF from the two different sources, hence the different values in properties. Impact strength estimated from notched Izod test, while the rest is obtained from notched Charpy test.

f Synthesized in our group by Weinland.^11^.

Monomers that are more flexible or are more capable of molecular motion, like CHDM and 1,4-butanediol, tend to be beneficial to the impact properties of polyesters. Our results align with this assumption: PICT and PIBT show significantly better impact performance in comparison to the other synthesized polyesters. CHDM is commonly studied due to its conformation changes and capacity for impact absorption. The conformations in CHDM shift mainly between two different chair structures, which may cause cooperative motions between adjacent repeat units. These movements will then force the terephthalate linkage to move, causing larger scale motion.^43^ These phenomena can be studied, for example, by comparing PCT and PET. This capacity to absorb energy for conformation changes, combined with CHDM's ability to perform other types of motions (rotational, translational), boosts the impact strength of materials. Since CHDM is one of the best diol candidates for high impact and high *T*_g_, it was chosen as co-diol to compare isosorbide-based terephthalate and furanoate polyesters (PICT and PICF).

The selected diacids, TPA and FDCA, behave differently in terms of motion and interchain interaction. The structure of terephthalic acid allows for two main modes of molecular motion: the phenyl ring flipping and the motions of the carbonyl groups.^44^ These can be studied by DMA, by analysing the secondary relaxation of these materials, which reflects the energy of the molecular motions below *T*_g_. FDCA, on the other hand, displays no ring flipping due to its hindered structured – high polarity and non-linear rotation axis.^45^ These motions are connected to a material's impact strength: a polymer with more capacity for molecular motion can absorb more impact energy, since this energy can be transformed into rotation, flipping, conformation shift *etc.4*^43^ Since terephthalic acid has an extra mode of motion (ring flipping), it should at least partially explain why PICT shows much higher impact strength than PICF, even at much lower MWs. Another factor that contributes to this difference is the polarity of FDC A due to its oxygen atom within its ring, which not only hinders intramolecular motion but also increases interactions between chains, packing them more strongly and thus hindering their (interchain) mobility. As a result, FDCA polyesters can perform very well in terms of tensile properties, but mostly have low impact strength.

In linear molecules like 1,4-butanediol, the relatively long chain has a greater capacity for translational and rotational molecular motions than shorter molecules like ethylene glycol or oxalic acid, and thus allow for better impact absorption.^46^ Increasing the chain length, however, may cause significant detriment to stiffness and *T*_g_, as the literature shows for cases of large amounts of 1,5-pentanediol and 1,6-hexanediol.^13^ Diethylene glycol has a different behaviour due to the presence of a highly polar oxygen atom in the middle of its structure, which increases interactions between chains and limits molecular motions, similarly to FDCA polyesters, therefore increasing tensile modulus and greatly decreasing impact strength. Another way to reach a balance of elastic modulus, impact strength and *T*_g_ is to use more co-monomers, as shown in the case of PI_40_CTF_10_, with 10% FDCA and 90% TPA. Replacing 10% of TPA with FDCA caused an increase in the tensile modulus (2000 MPa compared to 1850 MPa of PI_40_CT). The higher impact strength compared to PI_40_CT might be an effect of different processing/formation of samples and molecular weight, but regardless of this difference both compositions are clearly excellent for high impact applications. The *T*_g_ (∼133 °C) is some 10 °C lower than that of PI_50_CT, which can be useful in case processing or synthesis is hindered by an exceedingly high viscosity. For the case of PIsSu, by replacing some of the succinic acid with terephthalic acid (PIsSuT_25_, with 25 mol% TPA), only a slight increase in impact strength is achieved, although an increase in *T*_g_ and a reduction in modulus are observed. PIsSu and PIsSuT_25_ both display very low impact strength, which probably indicates that succinic acid is not a suitable candidate to uphold the aimed balance in properties.

The polymers synthesized in our group, when compared to the isosorbide-free homopolymers, show that the introduction of isosorbide into the chains may bring different benefits in different polyesters. For all materials, their crystallinity was hindered/eliminated and their *T*_g_'s were greatly increased by the addition of isosorbide – for each mol% of isosorbide added, the *T*_g_ typically increases 1 to 2 °C. For the case of PIPT, adding these levels of isosorbide seems to increase the impact strength of PPT significantly (from ∼4 t o ∼10 kJ m^−2^), likely influenced by the elimination of the polymer's crystallinity. For PCT and PBT an increase in their tensile moduli is observed to increase to values above 2000 MPa, which otherwise are relatively low (∼1500 MPa) in the homopolymers. For PBT, the increase in impact strength is significant for notched samples (from ∼9 to 17 kJ m^−2^), showing that isosorbide improved the homopolymer's resistance to crack propagation. The mechanical properties of PCF do not seem to benefit from the addition, only its *T*_g_ is greatly improved. PIT, on the other hand, shows very high *T*_g_ (188 °C at relatively low molecular weight, 205 °C reported^47^ in the literature) and difficulty in melting, which hindered its successful synthesis at high molecular weights. It is expected, however, that PIT's rigidity is very high at the cost of its impact strength, based on its preliminary results and by taking as reference polymers with 100% isosorbide content, like PIsSu.

Changes in mechanical properties due to varying compositions of isosorbide and co-diols are not very obvious in the range of 40–60% IS. The modulus of PIPT does not undergo a significant shift by changing either co-diol composition from 40 to 60%, possibly due to similarity in rigidity between propanediol and isosorbide, and these compositions show the same impact strength as well. At 50% of each diol, however, other effects may be relevant such as changes in heterogeneity of moieties within the chains, which could have caused the modulus to decrease and impact strength to increase at this specific composition. Furthermore, slight changes in thermal and mechanical properties may be attributed to the presence of the hydrated compound of isosorbide, 1,4-sorbitan, which is expected to cause branching in the chains.

The case of PICT shows that in this composition range the modulus can vary (1850–2100 MPa), while impact seems to be very high (>40 kJ m^−2^) for the whole range, with higher values than polycarbonate – 35 kJ m^−2^. According to our results, sample formation/processing plays a crucial part in accurately determining the impact strength of these materials, so one must be attentive in order to lower the chance of producing misleading data caused by high moisture, sample defects, low molecular weight, too few samples *etc.* These effects can also contribute to the visible standard deviations in elongation at break values, which are very sensitive to sample defects. These results show that this composition range (40–60 IS%) seems to produce similar polyesters in terms of mechanical properties and amorphous behaviour, with the most significant distinction being the increase in *T*_g_ with increasing isosorbide content.

Overall, the synthesized materials that performed the best were PICT and PIBT. Both outperform the goals of *T*_g_ above 100 °C and modulus above 2000 MPa, while PICT surpasses ABS in impact strength by ∼120% (42 *vs.* 19 kJ m^−2^) and PIBT (17 kJ m^−2^) is slightly worse than ABS. For these properties, PICT and PIBT show outstanding performance, which is rare in polyesters, and are likely suitable for applications for which ABS is currently used. Furthermore, PICT presents much higher *T*_g_ (∼142 °C) than ABS (∼107 °C) or polyesters like PCTT (*T*_g_ ∼110 °C). Other materials, such as PIPT, might be considered with the use of impact modifiers and/or by changing composition of co-monomers, with the potential downsides of decreasing their *T*_g_ and tensile modulus, besides adding more complexity (more components, often fossil-based) to the potential (chemical) recycling of these plastics. The presence of isosorbide and other bulky monomers like CHDM seem to create polyesters with considerable free volume, which in turn translates into high impact strength. Combining these monomers shows that low chain mobility can be achieved, observed by the high *T*_g_'s. The amount of isosorbide, however, must be limited by combining it with flexible monomers like CHDM or 1,4-butanediol, in order to guarantee that the polymer will not be too rigid and therefore present low toughness. For such balance, the range of total isosorbide in comparison with the diols should be around 40–60%.

### Thermal properties and stability

**Fig. 3:**
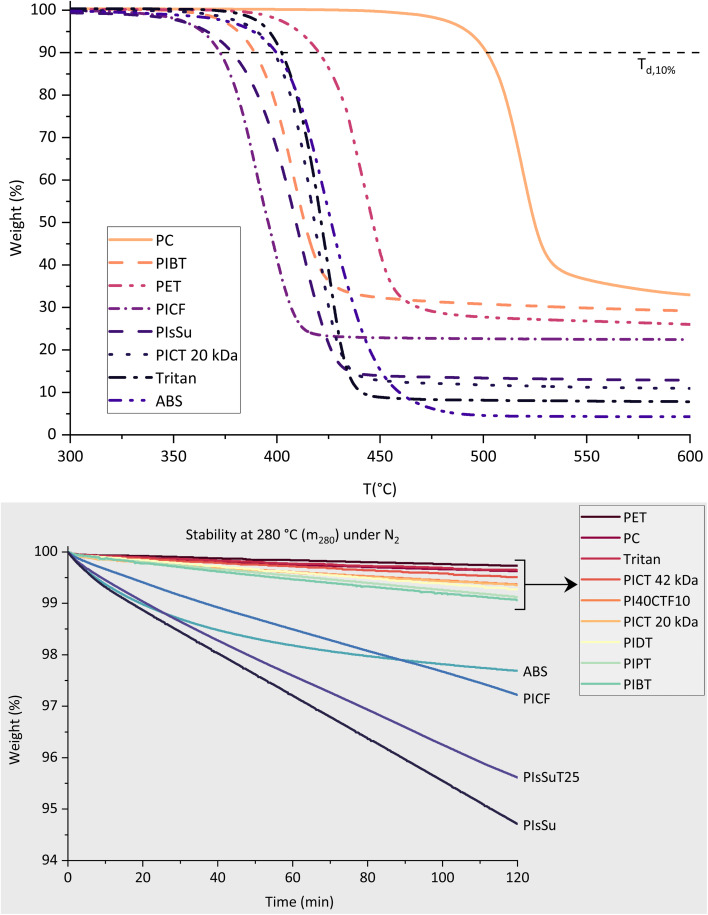
Thermal evaluation of polyesters and reference materials with TGA. TGA ramp at 10 °C min^−1^ at the top, and 2 hour isotherm at 280 °C at the bottom graph, all samples under nitrogen gas flow.

The thermal properties of the studied polyesters were evaluated with TGA and DSC (Table 3 and Fig. 3). The DSC scans were used only to obtain the *T*_g_'s of the polymers, given that the studied materials are all amorphous (like ABS) due to the significant amount of isosorbide, which is in accordance with literature.^16^ All materials show relatively high *T*_g_, above 90 °C, which is close to high performance polymers like ABS. The high glass transition temperatures are important for applications which require exposition to high temperatures – materials can be sanitized with boiling water for reuse, for instance, without deformation, which would otherwise occur in materials like PET (*T*_g_ ∼76 °C). Furthermore, having a high *T*_g_ is likely to slow down physical aging at typical exposure temperatures.^48^ The phenomenon of physical aging is undesired as it deteriorates impact strength and shape stability of polymers. Since the high *T*_g_'s of these materials are due mainly to the high percentage of isosorbide present, the synthesis method developed in our group may be a key element in developing high performance (*E*, *σ*) renewable polyesters with high *T*_g_.

**Table 3:** Thermal properties of the studied polyesters, analysed with TGA and DSC

Sample	Heating ramp @ 10 °C min^−1^, *T* = 25–600 °C					Isotherms (*t* = 2 h)			
	*M*_*n*_ (kg mol^−1^)	*T*_d_5%_ (°C)	*T*_d_10%_ (°C)	*m*_600_, residual mass (wt%) @ 600 °C	*T*_g_ (°C)	*T*_injection_ (°C)	*m*_N_2__ at *T*_injection_ (wt%)	*m*_air_ at *T*_injection_ (wt%)	*m*_280_ under N_2_ at 280 °C (wt%)
ABS	—	389	400	4.3	107	240	98.7	98.3	97.7
PET	—	407	417	23.0	80	280	99.7	97.6	99.7
Polycarbonate	—	492	502	33.0	142	280	99.6	98.7	99.6
Tritan	—	396	402	7.8	110	285	99.6	98.3	99.6
PICT 20 kDa	20.0	390	399	10.9	140	265	99.6	98.8	99.4
PICT 42 kDa	41.9	393	401	18.1	148	265	99.7	99.3	99.5
PIBT	17.8	381	389	29.2	107	220	99.8	99.6	99.1
PIPT	25.4	381	389	26.6	119	235	99.8	99.6	99.1
PIDT	22.3	390	401	27.1	91	215	99.8	99.7	99.3
PICF	37.7	364	373	22.4	129	240	99.4	99.4	97.2
PIsSu	43.9	366	378	12.8	80	185	99.4	99.3	94.7
PIsSuT_25_	20.2	369	382	25.9	102	185	99.5	99.4	95.6
PI_40_CTF_10_	42.0	383	391	24.3	133	280	99.4	98.6	99.4

The TGA scans provide an indication of the thermal stability of the studied polymers in terms of mass loss, which serves as a general profile but does not account for all forms of degradation. The results of the heat ramps indicate that most of the analysed materials are highly stable, with similar performance to ABS, *Tritan* and PET. These results are similar to those obtained for PEICT for instance.^23^ Polycarbonate is an exception – it exhibits extremely high stability at high temperatures, losing the same percentage of mass at temperatures ∼100 °C higher than the rest. The worst performing materials in this analysis were PIsSu, PIsSuT and PICF, which are expected to behave worse due to the lower stability of succinate and furanoate moieties. FDCA-based polyesters are sensitive to thermo-oxidative degradation,^49^ and succinic acid can decompose into succinic anhydride. The isotherms show similar trends regarding thermal stability.

The TGA isotherms were used to check the stability of these materials under typical polymer processing temperatures. At the temperature of injection moulding processing (*T*_injection_), under nitrogen flow, the presented materials seem to perform equally well, with mass loss below 0.6%, with the exception of ABS, which lost slightly over 1%. Some polyesters showed a slightly higher sensitivity to oxidative degradation, like PICT (20 kDa) and ABS, which indicated more mass loss under air. At 280 °C (N_2_ flow), however, we see more pronounced thermal instability from PICF (2.8% loss), PIsSu (5.3%), PIsSuT_25_ (4.4%) and ABS (2.3%) compared to the other materials (less than 1%).

Overall, the materials synthesized in this study seem to perform very well in comparison to commercial polymers like PET, *Tritan* and ABS. The least thermally stable polyesters are also the ones which are considered the most incompatible with the balance between *T*_g_, impact strength and tensile modulus: PICF, PIsSu and PIsSuT_25_. Further investigation should provide more insight into the thermal stability of these materials. This could include rheology studies of thermal degradation and physical aging studies. Rheology allows for the better understanding of loss of viscosity with heating and shear, and such loss correlates with a decrease in molecular weight, which is not clearly visible through TGA. Physical aging studies will allow us to understand better how these materials behave in the long run, in other words for how long the high performance (*E*, *σ*) is maintained for each material LEGO bricks and car parts.

## Conclusions

The synthesis method developed in our group using reactive phenolic solvents may become a key tool for producing polyesters that balance high *T*_g_ with relatively high stiffness (elastic modulus) and high impact strength. By using this method, our group developed high isosorbide content polyesters with high molecular weights that display good performance for the three targeted parameters: PIBT with 1,4-butanediol, PICT with 1,4-cyclohexanedimethanol and PIPT with 1,3-propanediol. For replacing ABS, or polymers like polycarbonate, other polar co-monomers like diethylene glycol and FDCA should likely be discarded for such delicate balance in properties, since their high polarity and stiffness tends to lead to low impact strength in their polyester polymers.

PICT and PIBT seem to be excellent candidates for high performance (*T*_g_, *E*, *σ*) applications while maintaining high thermal stability. PICT also outperforms ABS significantly in terms of impact strength and *T*_g_, while maintaining relatively high elastic modulus, at 2 GPa. In aspects like rigidity and *T*_g_, PICT outperforms the commercial *Tritan* (PCTT). Compared to polycarbonate, the *T*_g_ of PICT is practically the same for 50% IS, while achieving comparable impact strength and modulus. PIBT also shows a good balance in the analyzed properties, though slightly higher impact strength would be desirable for replacing ABS, for instance. These characteristics may place these materials among the best renewable polyester candidates, to our knowledge, for substitution of ABS, bisphenol-A polycarbonate and other high performance fossil-based polymers.

Polyesters composed of certain combinations of bulky monomers like isosorbide, CHDM and Tritan's monomer TMCBD, tend to display a relatively high *T*_g_, high impact strength and relatively high stiffness. The capacity of these monomers to disrupt the crystalline structure of the polyesters and maintain a significantly high free volume seem to contribute to high impact strength, while not allowing for great chain mobility, which would reduce the *T*_g_. Combined with an efficient synthesis route like the reactive solvent method, these monomers are valuable for compositions of tough, rigid, high *T*_g_ polyesters.

## Data availability

The data supporting this article have been included as part of the ESI.†

## Conflicts of interest

There are no conflicts to declare.

## Supplementary Material

SU-002-D4SU00294F-s001
