# Supplementary material for: The search for rigid, tough polyesters with high Tg – renewable aromatic polyesters with high isosorbide content
Source: RSC Sustain. 2024 Aug 1;2(9):2644–56. doi: 10.1039/d4su00294f (PMC11353682; doi:10.1039/d4su00294f)
Supplement: SU-002-D4SU00294F-s001 [file SU-002-D4SU00294F-s001.pdf]

Supplementary Information (SI) for RSC Sustainability.
This journal is © The Royal Society of Chemistry 2024

The search for rigid, tough polyesters with high T_g - Renewable aromatic polyesters with high isosorbide content

Bruno Bottega Pergher[a] , Daniel H. Weinland[a] , Robert-Jan van Putten[b] and Gert-Jan M. Gruter[*a,b]

Supporting information

a. Industrial Sustainable Chemistry, van 't Hoff Institute of molecular sciences, University of Amsterdam, University of Amsterdam, Science Park 904, 1098 XH Amsterdam, The Netherlands.

b. *Avantium N.V., Zekeringstraat 29, 1014 BV Amsterdam, The Netherlands;†*

* corresponding author: G.J.M.Gruter@uva.nl

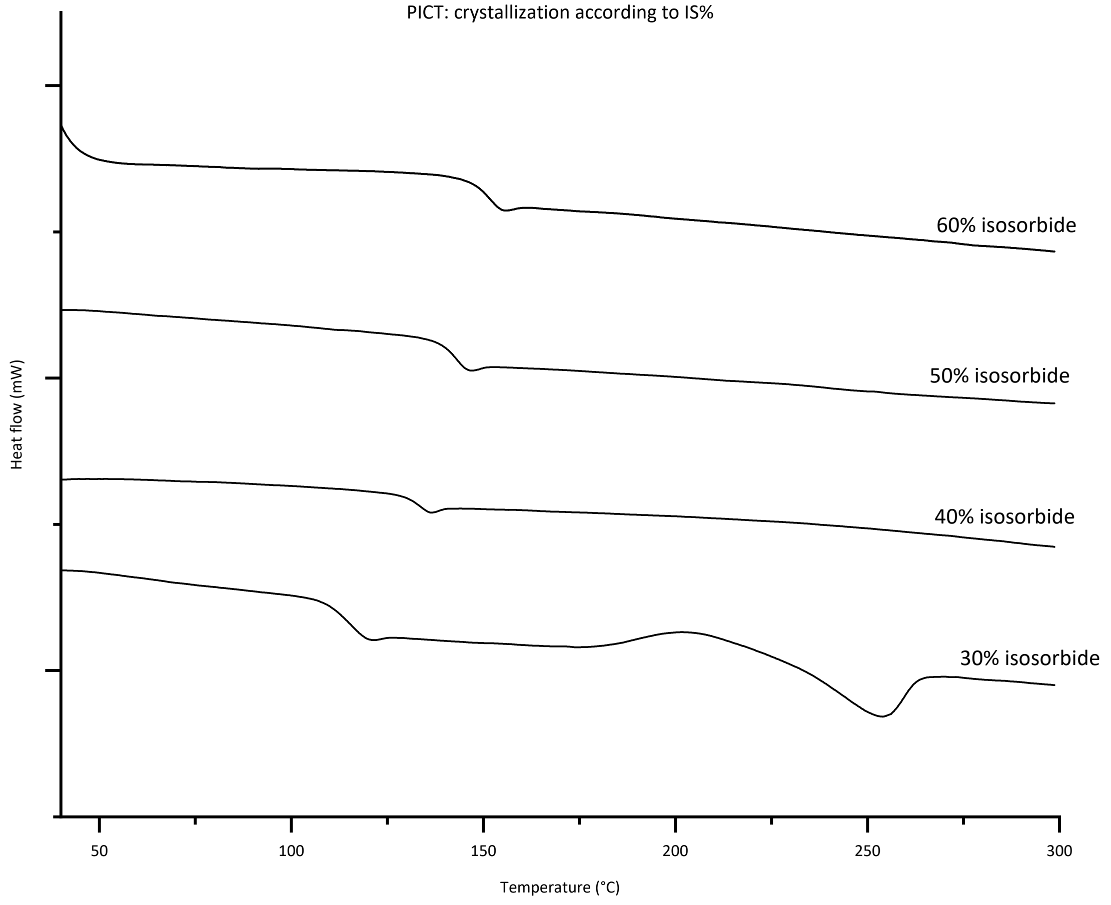

Figure 1 - DSC graphs of PICT with different isosorbide composition.

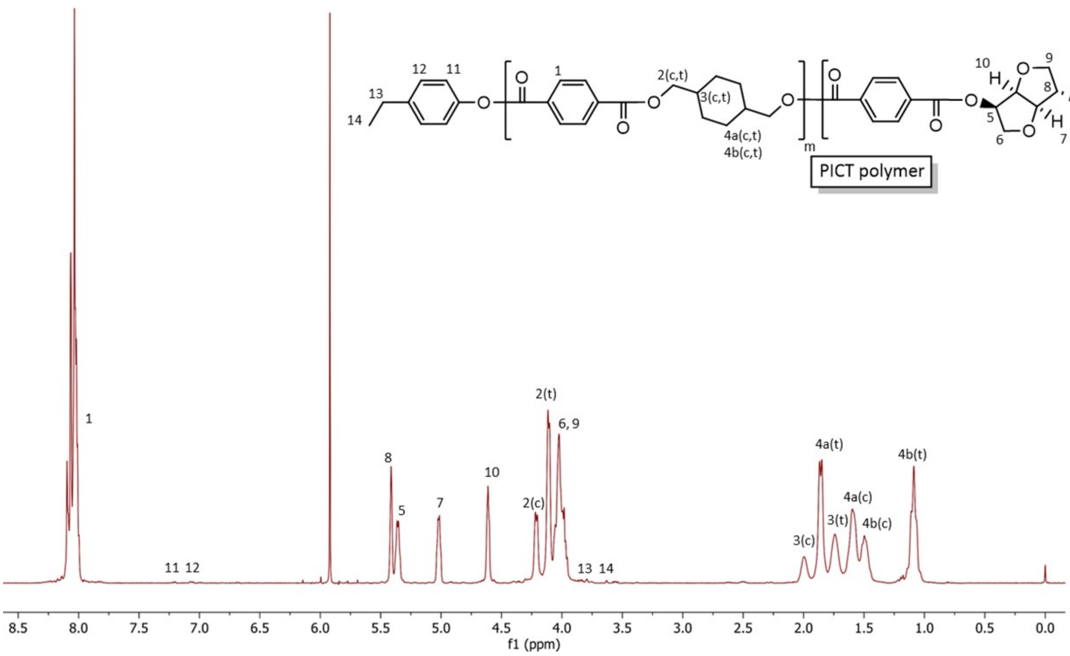
PICT polymer
1
2(t)
6, 9
8
5
7
10
2(c)
11 12
13 14
3(c)
4a(t)
3(t)
4a(c)
4b(c)
4b(t)
2(c,t)
3(c,t)
4a(c,t)
4b(c,t)
m
n
8.5 8.0 7.5 7.0 6.5 6.0 5.5 5.0 4.5 4.0 3.5 3.0 2.5 2.0 1.5 1.0 0.5 0.0
f1 (ppm)

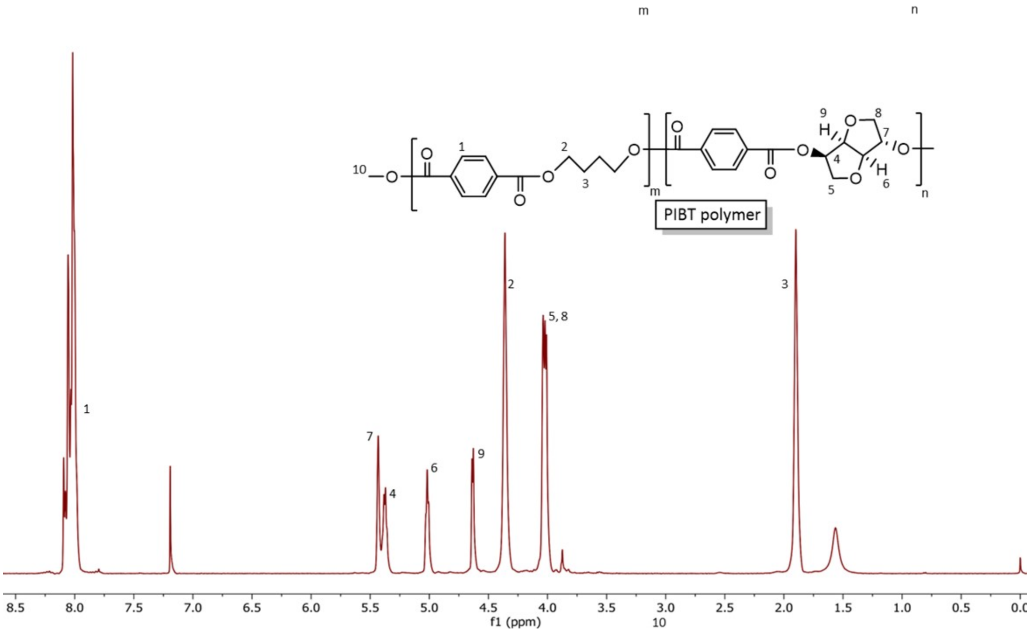
PIBT polymer
1
2
3
5, 8
7
4
6
9
10
m
n
8.5 8.0 7.5 7.0 6.5 6.0 5.5 5.0 4.5 4.0 3.5 3.0 2.5 2.0 1.5 1.0 0.5
f1 (ppm)

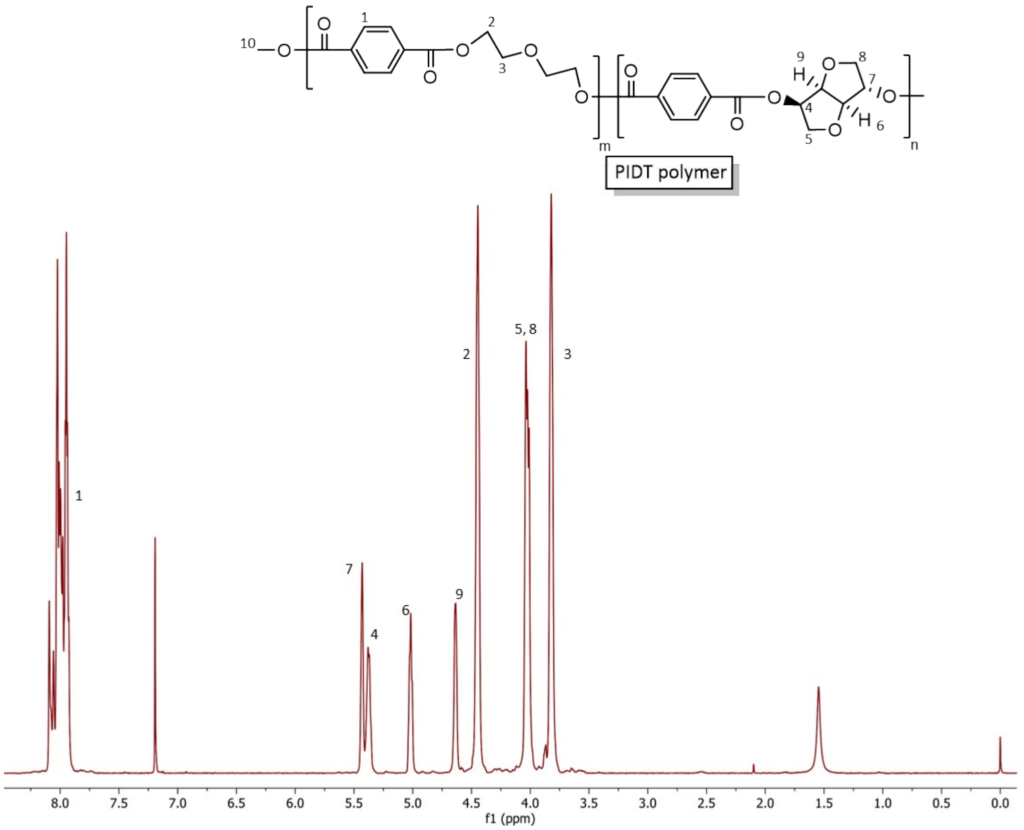
PIDT polymer
1
2
3
4
5, 8
6
7
9
f1 (ppm)
8.0
7.5
7.0
6.5
6.0
5.5
5.0
4.5
4.0
3.5
3.0
2.5
2.0
1.5
1.0
0.5
0.0

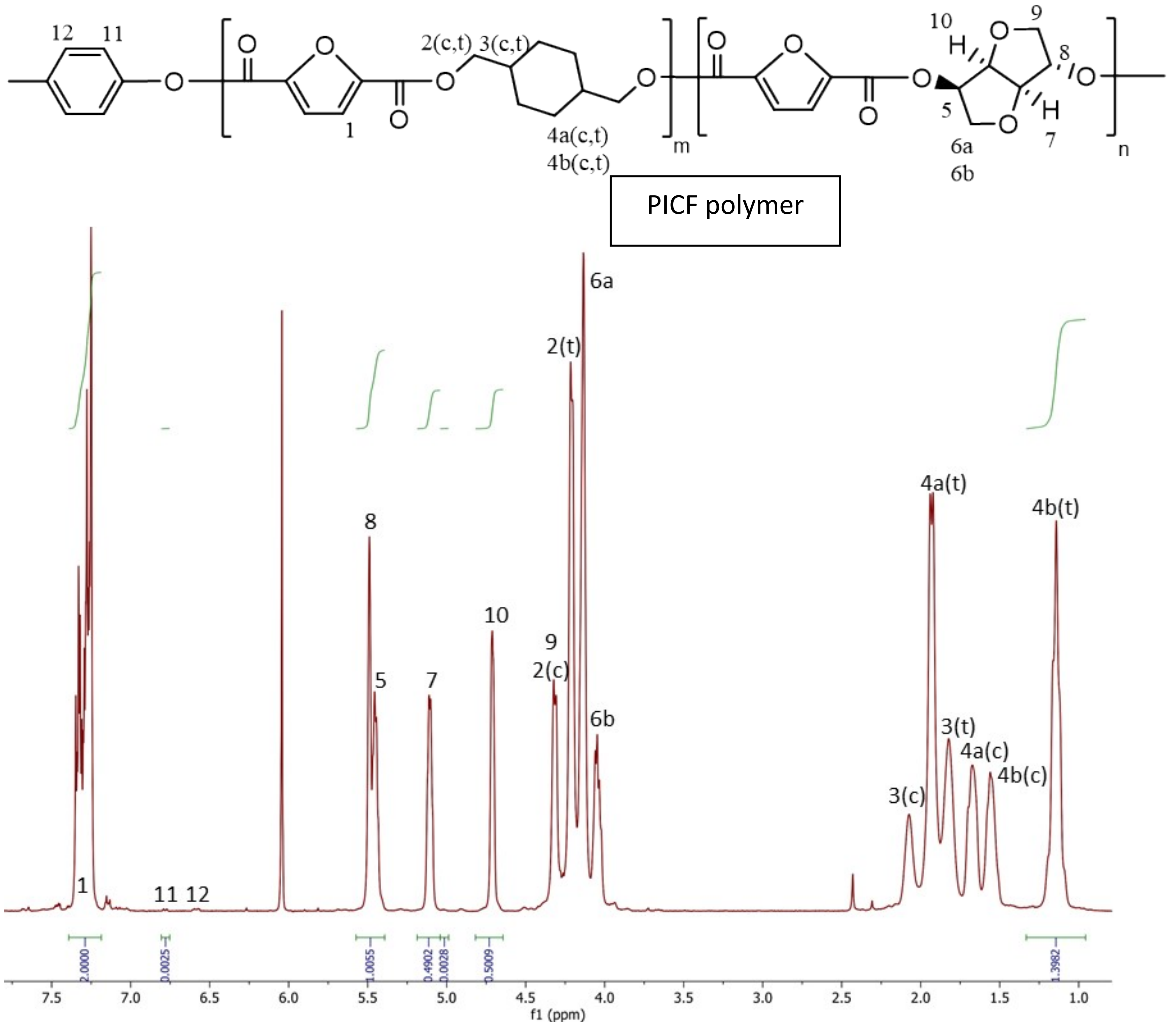
PICF polymer
12 11
2(c,t) 3(c,t)
4a(c,t)
4b(c,t)
6a
6b
1
11 12
8
5
7
10
9
2(c)
2(t)
4a(t)
4b(t)
3(t)
4a(c)
4b(c)
3(c)
2.0000
0.0025
1.0055
0.4902
0.0028
0.5009
1.3982
f1 (ppm)
7.5
7.0
6.5
6.0
5.5
5.0
4.5
4.0
3.5
3.0
2.5
2.0
1.5
1.0

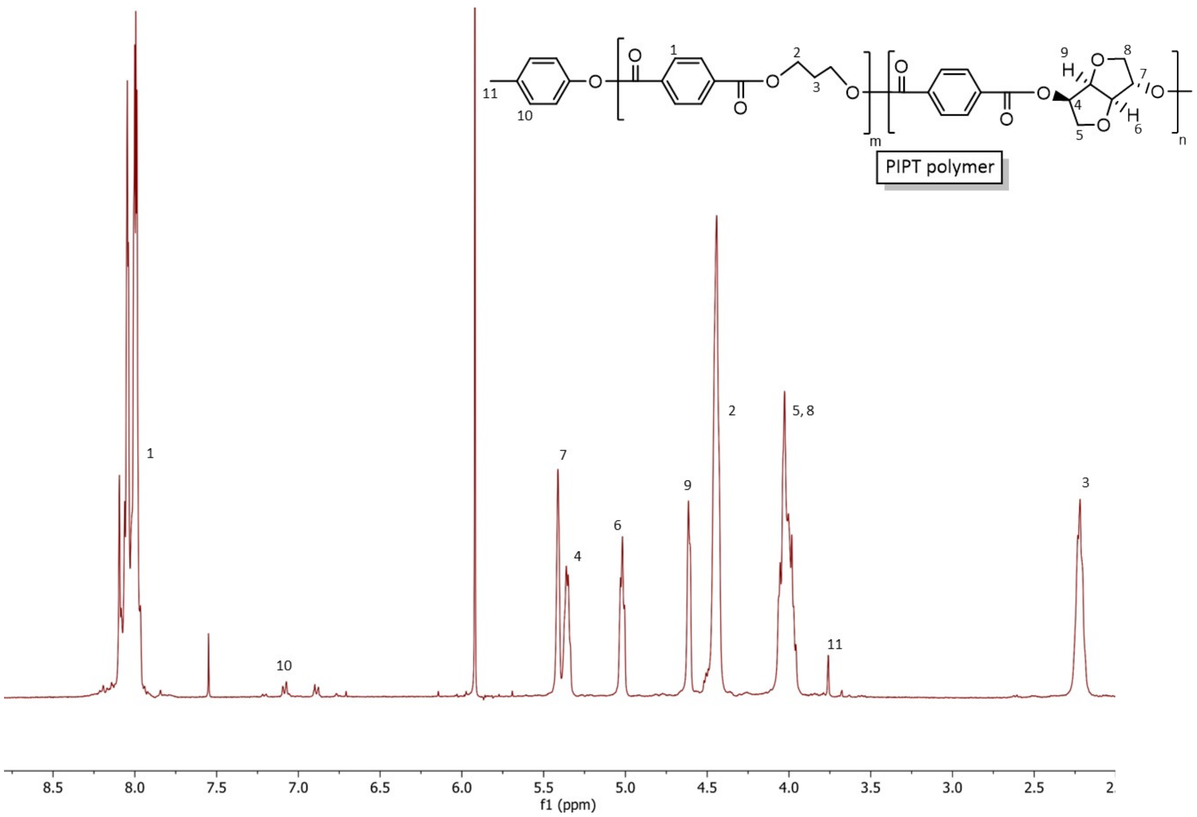

Figure 2 - Proton NMR of synthesized polyesters.

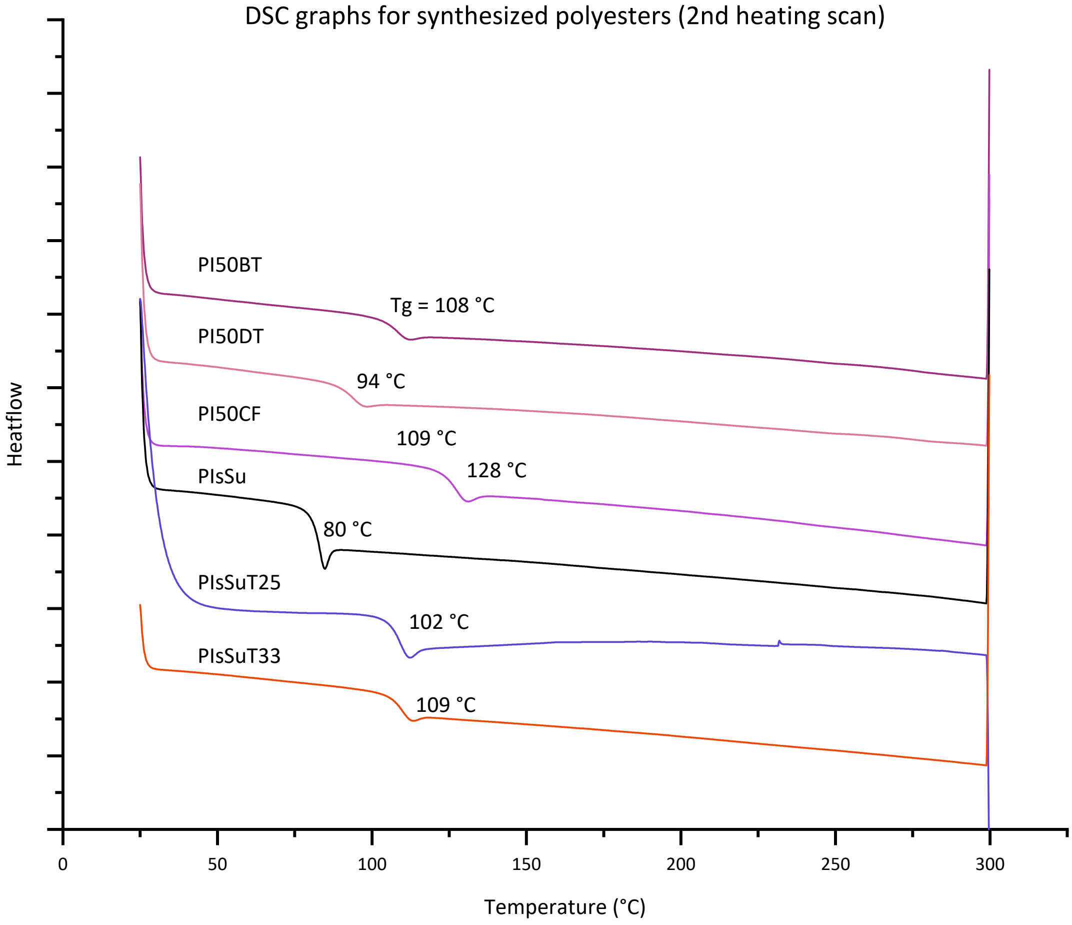

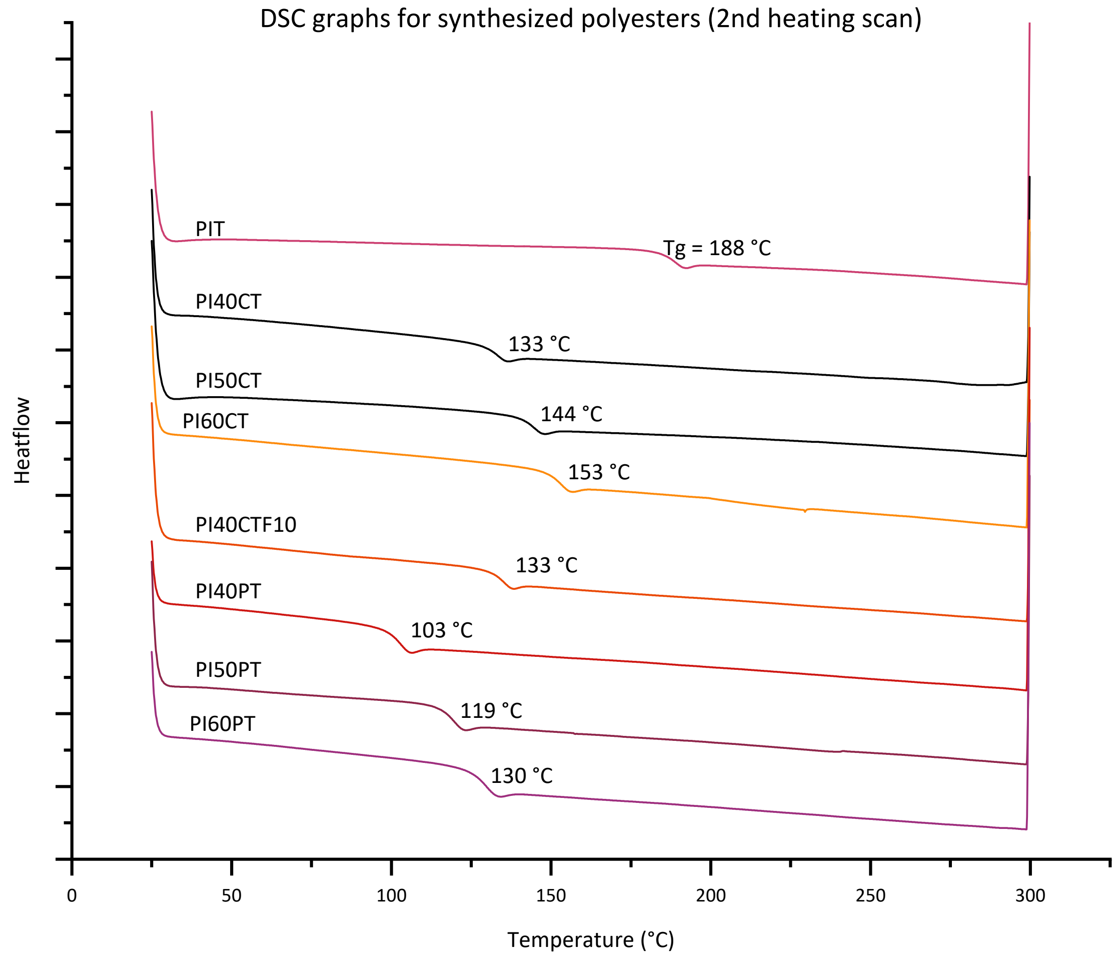

Figure 4 - DSC scans (2nd heating) for synthesized polyesters, with T_g indicated.

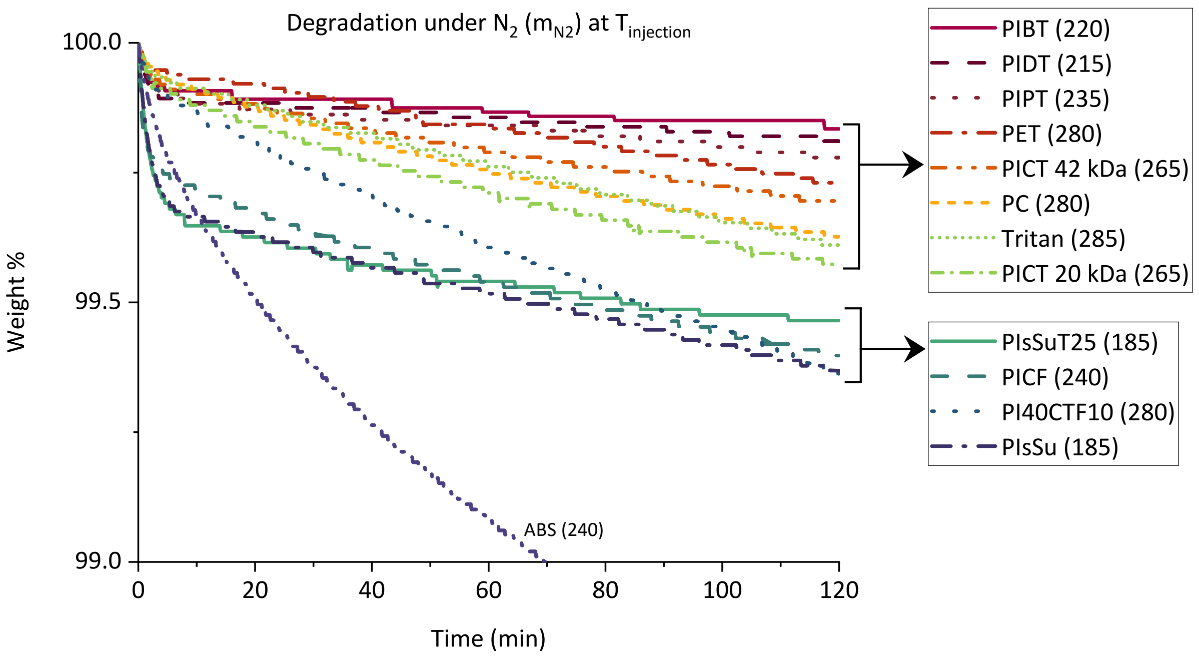

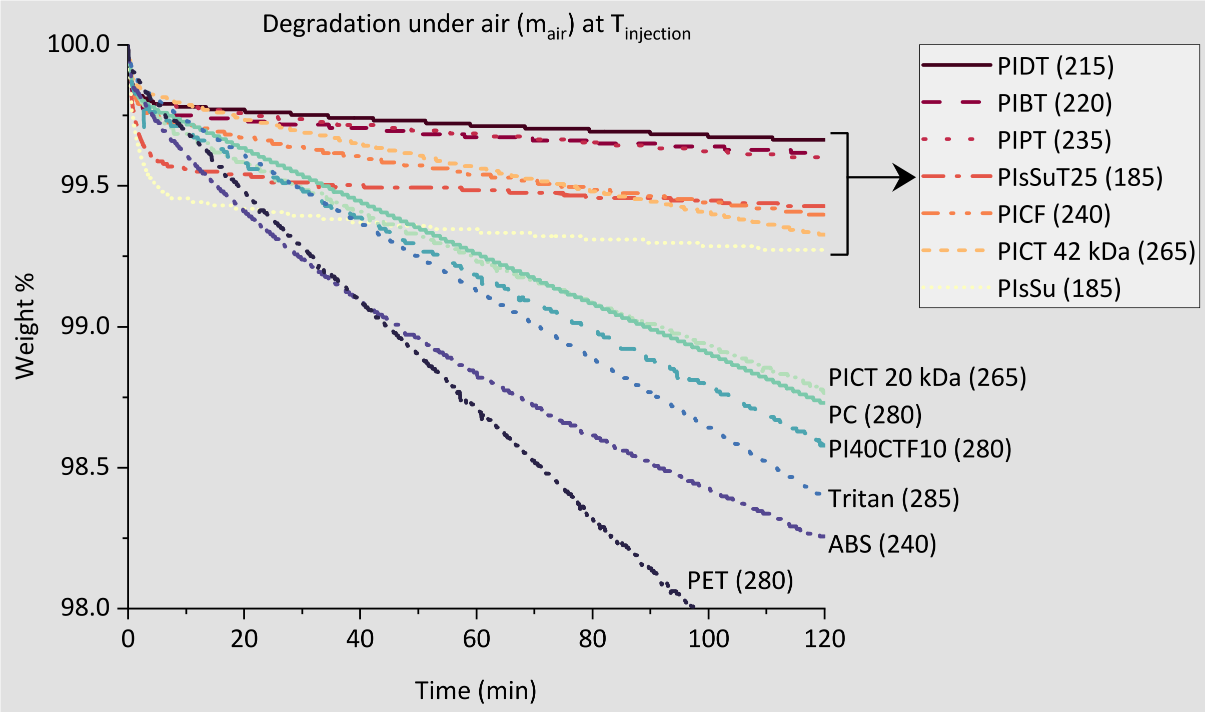

Figure 4 - TGA stability of samples under air (first graph) and nitrogen flow (second). Injection temperatures shown in brackets.

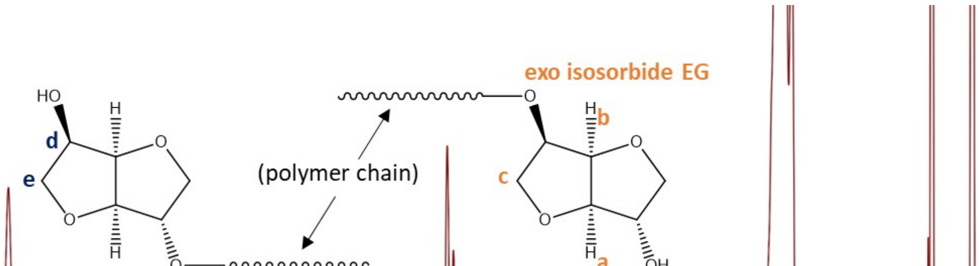

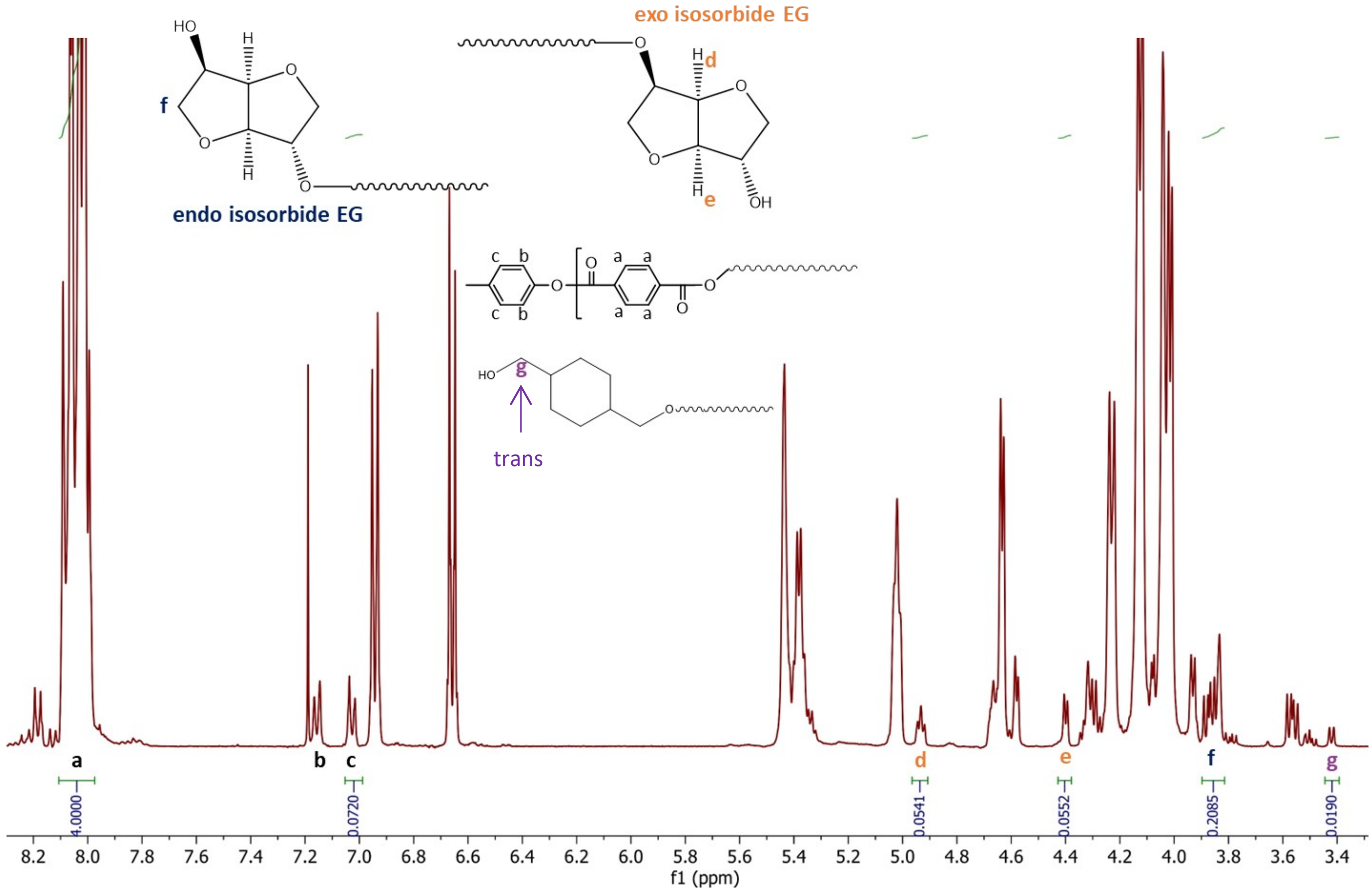

Figure 6 - End groups in PICT highlighted for calculation example. Same reaction as ^{13}C-NMR spectrum, in autoclave with 0.3 equivalent of p-cresol. Current time: 18 h of esterification (pre-vacuum). The integrals are normalized in comparison to the terephthalate group at 8.1 ppm, with 4 protons.This image can be used as an example of the calculations presented in the paper: peak ‘c’ has 2 protons, so a total of 3.60 mol% of end groups of p-cresol relative to the repeat unit. Analogously, ‘d’ and ‘e’ have 1 proton each, totaling ~5.4 mol% of isosorbide endo end groups and ‘f’ has two protons, which totals ~10.4 mol% of exo end groups – summing the two values, isosorbide end groups represent ~15.8 mol% compared to the repeat unit. At last, ‘g’ has 2 protons for the trans CHDM phase (70% of total), so in total 0.95 mol%/70% = 1.36 mol% total for CHDM.
